# Period product disposal in India: the tipping point

**DOI:** 10.1016/j.lansea.2023.100214

**Published:** 2023-05-13

**Authors:** Ashna Biju

**Affiliations:** a Gonville & Caius College, Trinity Street, Cambridge, CB2 1TA, United Kingdom; b School of Clinical Medicine, University of Cambridge, Cambridge, CB2 0SP, United Kingdom

## Abstract

The UN projects that India is now the world's most populous nation. However, with this rising population comes a significant need for safer period product disposal systems. Current practices include local incineration or burial of sanitary products, with harmful health and environmental impacts. This Viewpoint proposes a three-sphere model to develop long-term solutions for sanitary waste disposal in India, focussing on education, national organisation, and sustainability. Proposed solutions are developed and criticised with a consideration of why change has been limited thus far. A case study in Kerala is considered where all three spheres are incorporated with the help of a local non-governmental organisation, however, with limitations that could have been alleviated with national organisation. In effect, the Indian government must develop a centralised system for tackling sanitary waste disposal before a tipping point is reached.

As India is estimated to have overtaken China as the world's most populous nation,^1^ menstrual hygiene management and safe disposal of sanitary products is also becoming a growing concern. Even though 36% of India's 355 million menstruating women use sanitary pads,^2^ they are met with improper waste facilities, leaving poor outcomes for future generations. There is insufficient national coordination of sanitary waste collection, disposal, and transportation, which are compounded by social stigmas associated with menstruation.^3^^,^^4^ People who menstruate are then forced to develop their own strategies of disposing period products, with detrimental consequences. Products are thrown into nearby fields and unused wells; flushed down toilets; buried; or burned in people's backyards. More well-off individuals can afford to pay for private waste collection agencies, though it is still unclear where this waste is disposed. Despite inadequate sanitary waste management being publicly acknowledged, little is being done on a national level to solve this issue. Thus, this Viewpoint calls for change before a tipping point is reached.

Improper sanitary waste management has detrimental health and environmental impacts.^5^ Commercial sanitary pads are disposable and nonbiodegradable, potentially taking up to 800 years to break down into microplastics. Marine ecosystems are altered when organisms ingest these microplastics, and elevated levels of microplastics build up in food chains.^6^ When flushed in toilets, super-absorptive materials like polyacrylate absorb water, resulting in sewage backflow. This poses its own health problems for individuals living near water pipelines and canals. When deodorised products are buried, organochlorines in the products disturb the soil microflora. Moreover, menstrual blood infected with HIV or hepatitis retain their infectivity in soil, risking water safety.^5^ Conservancy workers unblock water systems often without proper protective equipment, exposing themselves to toxins and pathogens. Household-level incineration of waste is marginally better, however, burning of inorganic material releases carcinogenic dioxins which are persistent organic pollutants. Overall, safe period product disposal is essential for health and the climate.

This Viewpoint proposes a three-sphere model for solving period product disposal involving education, organisation, and sustainability, shown in Fig. 1. Each of these three spheres will be explored in detail, with an evaluation of challenges to implementing this model (perhaps explaining why change has been limited in India so far).

**Fig. 1:**
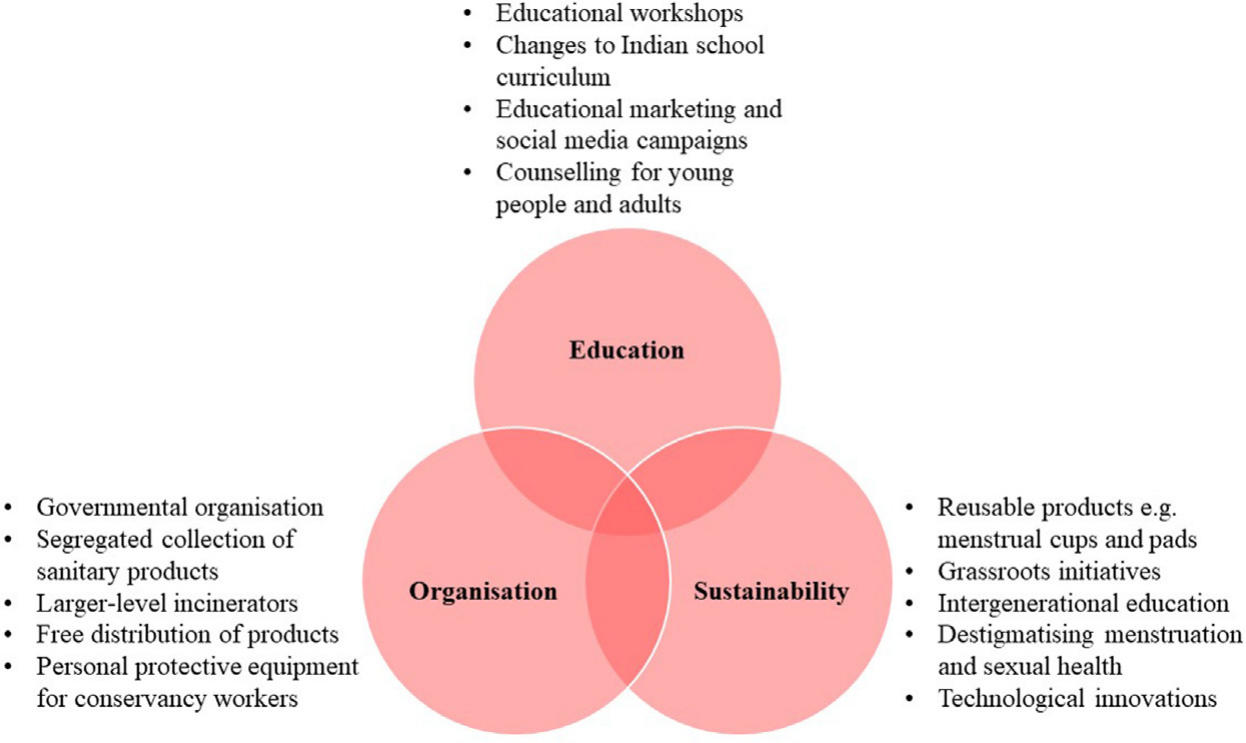
Proposed three-sphere model for tackling improper period product disposal.

First, professionals and social workers could provide education on menstrual hygiene management to young girls and boys, especially focussed on tackling social stigmas around periods. Stigma is deeply entrenched, and is influenced by cultural, religious, and patriarchal values.^4^ Indeed, in many parts of India, there is a long history of perceiving menstruation as an impure phenomenon, restricting women on their periods from cooking, bathing, praying and even entering the main part of the house.^3^ Annual educational workshops on menstrual hygiene management could be incorporated into the national school curriculum with endorsement from the Ministry of Education. It is important to make this available not just to girls because this helps destigmatise menstruation and provides education for people who menstruate but do not identify as women. This education should extend to adults in the community, with financial or social incentives for engagement. Moreover, primary care physicians could do more to educate patients on menstrual hygiene management and treat them holistically, especially as members of a trusted profession.^3^ Overall, educational solutions must originate at a root-level to ensure long-term change.

Second, reusable sanitary products should be encouraged as a more sustainable alternative.^7^ Menstrual cups and reusable cloth pads can last from 2 to 10 years, depending on manufacturer and customer use. Benefits of such products include reduced usage and unsafe disposal of commercial sanitary pads, reducing the health and environmental impacts detailed earlier. However, reusable period products are not widely used in India. They are initially more expensive to buy (though cost-effective in the longer run) and are not as widely advertised or sold. There are fears around insertion and removal of menstrual cups breaking the hymen, with many women worrying they may ‘lose their virginity’ (virginity culturally associated with intact hymen).^7^ These beliefs could be challenged with educational workshops destigmatising sexual health and virginity, as detailed earlier. Currently, such schemes are not prevalent and would require significant financial and governmental support. Indeed, all these factors may contribute to the limited advertising, marketing, and sales of menstrual cups in India.

Third, the national government must take initiative to organise safe sanitary product disposal. The onus should not exclusively be on individuals, local charities, or non-governmental organisations. A centralised system is needed to create sustainable solutions for the long-term. This could be aided by existing guidance from international health organisations such as UNICEF.^8^ The co-operation of several Ministries in India would be needed for a large-scale effort to solve this problem, including the Ministry of Women & Child Development and the Ministry of Health & Family Welfare. An effective strategy would be to reward or penalise period product manufacturers who play a significant role in influencing consumer habits. For example, Niine Sanitary Napkins provide biodegradable bags with their products, and have also been partnering with schools to install incinerators. In 2021, the Minister of Environment, Forest and Climate Change made it mandatory for all manufacturers to provide such biodegradable bags, however, there is little evidence on how this has been received.^9^ Furthermore, there is limited incentive for the government to improve sanitary disposal systems due to a lack of academic literature to indicate demand. This, in turn, leads to an insufficient push for research into menstruation, resulting in a negative cycle of inaction. This is compounded by social stigma to which members of government are just as susceptible as the public. Overall, despite recent recommendations from the Central Pollution Control Board in India to improve sanitary waste disposal,^10^ they are not sufficiently implemented. Further research into the barriers preventing this may encourage the Indian government and period product manufacturers to take this issue seriously.

A case study where the three spheres are considered is in Muhamma, a village in the Alappuzha district of Kerala, hoping to be the first synthetic pad-free village in India.^11^ In 2019, the village council distributed 5500 cloth pads and 500 menstrual cups, for a subsidised rate with the help of a non-governmental organisation. This was supplemented with awareness campaigns teaching people how to use these reusable alternatives, and the importance of them.^11^ However, there are limitations to this initiative as a model case study as no follow-up has been conducted to study the villagers’ continued use of reusable period products. This initiative would also have benefitted from increased funding and national organisation to improve accessibility for individuals who could not afford the subsidised rate, by instead distributing products for free.

According to the UNICEF theory of change for menstrual health management, the lack of supportive systems for waste disposal is a significant barrier to equitable outcomes in any country.^8^ In India, though local schemes aim to tackle sanitary waste disposal, they are limited by funding and research, and more awareness and organisation is needed on a national level. The Indian government must take charge to develop long-term solutions for sanitary waste disposal, based on the proposed three-sphere model. A centralised system is needed for national change, and this must be done before a physical, and metaphorical, tipping point is reached.

## Contributors

AB conceptualised and wrote the paper.

## Declaration of interests

None.
